# The algorithmic self: how AI is reshaping human identity, introspection, and agency

**DOI:** 10.3389/fpsyg.2025.1645795

**Published:** 2025-07-11

**Authors:** Jeena Joseph

**Affiliations:** Department of Computer Applications, Marian College Kuttikkanam Autonomous, Kuttikkanam, India

**Keywords:** algorithmic self, AI, identity, introspection, personal agency, ethical implications

## Introduction

Artificial Intelligence (AI) is no longer a mysterious technological presence hiding behind screens; it is intertwined with the most intimate dimensions of who we are. From playlists on Spotify to language model-generated replies, personalized news feeds, to self-tracking wellbeing apps, algorithms co-create the way we know ourselves and belong in the world today. In spite of the worldwide love for AI for its revolutionary utility in various sectors, it is necessary to examine how it exerts a subtler, but no less impactful, psychological impact on personal self, self-awareness, and agency (Banja, 2020; Namestiuk, 2023). This article examines the idea of the “Algorithmic Self”—something that indexes how AI's interpretive feed starts mediating self-knowledge, not merely shaping what we do, but also who we become and the narratives we narrate to ourselves.

The “Algorithmic Self” refers to a form of digitally mediated identity in which personal awareness, preferences, and even emotional patterns are shaped through continuous feedback from AI systems (Turtle et al., 2024). It is not merely a self-reflected in technology but co-constructed by it—where algorithms do not passively reflect the self but actively participate in its formation (Masiero, 2023). This concept draws loosely on post-humanist and surveillance capitalism frameworks, which describe the self as increasingly entangled with and constructed by digital infrastructures (Bartley, 2019; Leander and Burriss, 2020). In this view, the self is no longer autonomous and inwardly derived, but assembled across interfaces, platforms, and predictive logics.

The more time we spend in a world where algorithms dictate so much of what and how we know, the more necessary it is that we explore how AI reconfigures not just what we are doing, but who we are doing it as (Wang, 2023; Fesce, 2024). The “Algorithmic Self” is the place where human consciousness encounters machine feedback—a frontier that is both emancipating and limiting (Canbul Yaroglu, 2024). This article is intended to shed light on how algorithms construct identity, and a critical exploration into the psychology and morals in a world fast becoming driven by AI narratives. This exploration unfolds through a series of interconnected lenses, each illuminating how artificial intelligence mediates different aspects of selfhood in the digital age. Though each theme addresses a distinct psychological or existential concern, they collectively depict a broader transformation—one where the boundaries between human introspection and algorithmic feedback blur. Rather than presenting isolated observations, this article seeks to build a layered understanding of how AI co-produces identity, emotion, and agency.

## From mirror to mold: the emergence of the algorithmic self

In the past, the evolution of human identity has occurred in conjunction with society, family dynamics, cultural narratives, and introspective self-reflection. Identity was determined by how we engaged with others, cultural practices, and even periods of solitude and self-reflection. In the digital era, however, identity is being determined through engagement with algorithmic systems (Lee, 2025). Recommendation algorithms, predictive language models, and behavior-monitoring AI capabilities now not only present us with what we might consume but with how we ought to feel, how we ought to think, and even how we ought to self-categorize (Ferrara, 2024). The formerly passive digital screen is today a participant in shaping the self (Brubaker, 2020).

The “Spotify Wrapped” trend is a prime example: fans eagerly anticipate the algorithmic summaries of what they listened to in the previous year—as if Spotify knows them better than they know themselves (Annabell and Rasmussen, 2024). In the same manner, mood-tracking apps forecast when a user is likely to be anxious, and smartwatches prompt wearers toward “improvements” in behavior based on biomarker data. These apps and numerous others not only serve to mirror the behavior of the user but also to define, shape, and control the user's sense of self over time (Schueller et al., 2021).

A particularly illustrative case of algorithmic identity shaping is Spotify Wrapped, an annual “algorithmic event” that repackages users' listening behavior into curated data stories (Annabell and Rasmussen, 2024). Presented in a format resembling social media stories, these summaries are widely shared, often perceived not just as records of behavior but as reflections of personal identity. In a creative workshop study, Annabell and Rasmussen found that users often critically engaged with these algorithmic representations—sometimes feeling seen, other times unsettled—yet many accepted the summaries as accurate or insightful accounts of who they are. This illustrates how commercial platforms use data not only to serve users but to define them. Brubaker extends this view by arguing that hyperconnected digital infrastructures do not just reflect the self but participate in governing it—by framing how we see ourselves, behave, and are categorized by others (Brubaker, 2020). Together, these studies reveal how algorithmic selfhood is co-constructed in moments of both compliance and critical reflection.

In this algorithmically mediated world, self-knowledge is not a reflection or discovery experience, but rather a reflection experience, one that is external and that is facilitated by interpretations from machines. AI is not just a passive witness; it is a mirror that not just mirrors the self but shapes it in conformity with algorithms. As the algorithm begins to play an increasingly prominent role in shaping identity, it also begins to influence the cognitive tools we use to understand ourselves. The same systems that define our preferences and behaviors now offer interpretations of our moods, thoughts, and intentions—potentially shifting the practice of introspection from a personal, reflective act to an externalized, data-driven summary. This transition raises deeper questions about how much of our inner world we are outsourcing.

## The collapse of introspection: delegating inner work to AI

Though AI brings efficiencies and insight, it also quietly substitutes essential cognitive processes such as introspection and self-inquiry. Self-awareness, traditionally developed through practices like journaling, meditation, or therapy, is outsourced to algorithmic systems that provide predetermined summaries of one's moods, behavior, and thoughts (Lang, 2024). The rise of emotionally intelligent AI solutions, such as chatbots and therapeutic apps, has given rise to a system in which users depend on algorithms for emotional navigation, moral decision-making, and even self-reflection (Spytska, 2025).

This delegation is not in itself problematic. AI systems can assist by providing insight or helping with emotion regulation. But problems can arise when introspection is outsourced completely. Users can begin to trust the algorithm's reading of things over their own feelings, intuitions, or recollections. Excessive use of AI-assisted learning resulted in cognitive disengagement and poorer memory retention (Bauer et al., 2025). The same degradation can occur in emotional self-awareness: the greater that individuals allow machines to read them, the less they acquire the subtle, critical self-awareness that results from engaged reflection and personal insight.

When AI assumes responsibility for “emotional reflection,” individuals can become estranged from the richness of their own emotional lives (Currie et al., 2025). What this poses are not the loss of introspection, but a change in how individuals relate to emotions. Outsourcing emotional intelligence to machines can, in the long run, produce a diminished sense of personal emotional awareness and make it difficult to negotiate subtlety in emotions without the help of the machines.

At the same time, it is equally important to recognize the potential for AI systems themselves as powerful tools for expanding introspection and personal agency when utilized carefully (Sackett et al., 2024). Apps like AI-enhanced journaling assistants, tracking platforms for emotions, and AI mental health programs like Woebot or Wysa offer new modalities for the individual to discern and regulate their emotional states (Beatty et al., 2022; Darcy et al., 2023; Inkster et al., 2023). For some users, technology like this fosters deeper self-consciousness through the offer of structured prompts, reflective summaries, or even therapeutic conversations that would not otherwise be accessible (Dinesh et al., 2024). In a word, AI can potentially complement human introspection, supporting rather than replacing it—should the user be careful with regard to the lines between facilitation and delegation.

When individuals grow accustomed to receiving emotional and cognitive feedback from machines, their decision-making processes can subtly shift as well. What begins as assistance often turns into direction, as algorithmic suggestions are taken not merely as options, but as defaults. Over time, these patterns shape not just what people do, but how they conceive of choice, preference, and volition itself.

## Predictive personalization and the illusion of choice

One of AI's best-known virtues—predictive personalization—also carries secret costs to the psyche. Algorithms screening content in accordance with previous behavior promise to make decision-making easy and offer targeted suggestions (Kumar and Uchoi, 2025). As content optimization has pragmatic advantages, it limits the variety of choice by curating the information itself and the situations in which decision-making occurs. Predictive algorithms not just offer suggestions for what we may like; they define what we like, what we prefer, and how we act by constantly reinforcing what we have already expressed interests in (Boff Medeiros et al., 2025).

Take predictive text apps, for example, that not only fill in sentences but, in doing so, also reshape the intent and tone behind the original message. A writer wishing to say something with emotional depth might end up with a generic, bland, or overly formal message due to the algorithm guessing what is socially acceptable from previous use. Eventually, this ongoing input can homogenize expression, stifling individuality and suppressing a person's sense of authentic expression in communication.

In addition, if the users constantly accept the suggestion provided by the algorithm, they might end up confusing choice with consent. This behavior, also known as “preference reinforcement” in the field of behavioral economics, can cause cognitive entrenchment, as individuals end up perceiving that their preferences are inevitable or predetermined—when they are actually constructed to some extent by the algorithms themselves (Chen et al., 2025a). The illusion of choice, therefore, becomes a weak but potent force in determining personal identities.

These small, seemingly inconsequential decisions accumulate, producing recursive effects. As algorithms continue to respond to and reinforce particular patterns of engagement, individuals may find themselves locked into digital mirrors that reflect only parts of who they are. In these feedback systems, self-perception may harden into a fixed identity, even when the human self remains inherently dynamic and evolving.

## Digital identity feedback loops: entrenching self-perception

AI systems tend to run in closed loops, reinforcing the content they arere being trained with. Carried over to personal identity, such feedback loops can solidify self-concepts in a manner that can hinder personal evolution. If a user is mostly engaging with content that is termed “introvert” or “low-energy,” for example, recommendation systems can provide similar content in return, trapping the user in a digital echo chamber of self-perception, reinforcing a limiting self-image (Chen et al., 2025b).

These identity feedback loops replicate the psychological risks that clinical labels present—where diagnoses like “depression” or “anxious” can become self-fulfilling (Schlozman et al., 2025). In the digital sphere, instead, the labels occur through inference by algorithms, not clinicians, and the effects on self-concept may be imperceptible. The user might not realize that this shaping is occurring. The risk is in reinforcing that label without leaving space for dynamic alteration or contradiction. The algorithm is aware of the things you engage with, but not who you might become.

## The quantified self: from subjective experience to numerical identity

The expansion of the “quantified self” movement—in which individuals quantify everything from sleeping patterns to emotional states using AI-based instruments—reflects a mounting fascination with data-based identity (Good and Horn, 2025). Self-tracking can encourage consciousness and even self-refinement; but it can also reductively strip away the messy, the intuitive, and the subjective that distinguish us as human beings (Spence et al., 2025).

For instance, someone who wakes up and looks at their sleep score can become accustomed to judging the quality of the entire day from that single number, without regard for how they really feel. The self gets reduced to a quantified self, undermining a sense of internal, bodily experience. In the long term, the repeated compulsion to “improve” one's actions can take on the form of a hyper-surveilled self in which one is reduced to being judged according to measurable outputs—little different from corporate performance metrics (Tan, 2025).

This self-monitoring also causes anxiety, especially when one is not reaching the “optimal” performance or output levels required by such systems. The need to maximize one's acts on the basis of metrics can also lead one to reject one's own experience or sense in favor of digital evidence, leading to a discrepancy between experience and digital judgment.

## Emotional delegation and the rise of sentiment-aware AI

AI is being programmed to read and react to emotions through sentiment analysis and natural language processing. Therapeutic AIs such as Woebot and emotional chatbots such as Replika seek to offer emotional support by being comforting, validating emotions, and walking users through difficult emotions (Boucher et al., 2021; Darcy et al., 2023). They democratize mental health resources yet also make emotional delegation, or letting machines handle or adjust our emotions, become normal.

The emergence of sentiment-aware AI is fraught with a potentially serious risk: emotional conditioning. Users might begin to tailor the display of emotions to comport with the AI's expectations, taking on its “emotional logic” driven by patterns as opposed to true human empathy. Eventually, this could produce emotional conformity—whereby users display emotions that fit with machine expectations rather than with the true emotion they are experiencing (Tan and Jayasekara, 2025). Instead of building emotional intelligence, emotionally intelligent AI could be promoting emotional uniformity.

## The erosion of narrative agency

According to narrative psychology, humans make sense of ourselves through the narratives we narrate in our lives. These narratives provide for meaning-making, continuity, and coherence. In times of algorithmically curated reality, however, the narratives that we narrate to ourselves become ever more determined by those generated by machines (Reid, 2024). Events in our lives become structured and narrated not through introspective narratives, but through algorithmically culled highlights—Instagram highlights, fitness milestones, digital memory cues, etc.

Here, the AI becomes a co-author to one's story of self. But it does not capture the mess, the contradiction, that gives human narratives meaning. If we recount our digital lives in well-tuned, optimized chunks, it can flatten the richness of what we experience and prevent psychological integration. Growth, resilience, and self-definition are processes that need contradiction, change, and ambiguity—all things that algorithmically edited stories often miss (Ciszek et al., 2025).

The psychological dimensions of this transformation are profound, but they also carry significant ethical weight. If identity, emotion, and even narrative are increasingly mediated by opaque systems, questions arise about authenticity, authorship, and autonomy. The implications stretch beyond individual experience, pressing society to reconsider what it means to live a self-determined life in an algorithmically optimized world.

## Ethical and existential implications of the algorithmic self

The Algorithmic Self is not just a psychological change; it raises serious ethical and existential implications (Lagerkvist et al., 2024). What does it mean to “know oneself” if a lot of that “knowing” is being done by machines? Who does the version of you that the algorithm has constructed belong to? Can a self be authentic if it is being optimized by invisible code on a continuous basis? These questions resonate with existential psychology and AI ethics. As AI becomes more entrenched in everyday experience, it is imperative that individuals, educators, designers, and clinicians ensure algorithmic literacy—the capacity for self-reflection on how AI is mediating perception and selfhood (Andreescu, 2025). Without that literacy, individuals become susceptible to being shaped by outputs from machines rather than intentional self-authorship.

The rise of the Algorithmic Self presents a profound ethical dilemma at the intersection of autonomy and algorithmic determinism. As AI systems increasingly shape our preferences, decisions, and self-perceptions, the individual's sense of authorship over their own life may erode. This challenges the notion of a self-directed, autonomous person and raises concerns about the subtle influence of algorithmic nudges on human agency (Calvo et al., 2020). Moreover, these systems are not neutral; they are shaped by commercial interests, cultural assumptions, and underlying data biases. The construction of digital identity thus becomes entangled with broader questions of power, representation, and fairness (Mhlambi and Tiribelli, 2023; Aizenberg et al., 2025). Addressing these implications requires not only technical safeguards but also an ethical and socio-political reckoning with how AI mediates the very fabric of personhood.

## Reclaiming psychological agency: the path forward

To offset the passive exposure to algorithmic feedback, individuals need to engage in active self-construction. This entails the development of digital habits that prioritize reflective awareness, diverse media consumption, and the scrutiny of AI recommendations (Kohn, 2024). By being aware of algorithms, consumers can reclaim control over the way they use AI-generated content.

At the design level, AI systems need to adapt to augment, not replace, introspection by humans. Developers can implement features that promote composing personal interpretations of data, inserting time delay for reflection, or including counterarguments to interrupt preference loops. By doing this, we can design AI systems that enable individuals to retain their narrative agency as opposed to limiting their potential.

Schools and educational systems can assist by integrating digital selfhood in curriculums, educating scholars on how to identify and cope with the psychological effect of AI. Therapists can also bring up discussions on digital identity in therapy, walking clients through the emotional ramifications of AI-facilitated living.

## Conclusion

AI is not just a tool—it is becoming a co-author of the self. As algorithms inform our tastes, read between the lines, and predict what we might do next, they redefine what it means to be self-aware. The Algorithmic Self is both promise and peril: it can heighten self-awareness or dismantle introspection and agency. As we proceed, we must make sure that AI works for the self—and not the reverse. Taking back psychological agency from the age of algorithms is possible, but it entails careful reflection, careful design, and a dedication to maintaining human authenticity. In a future with ever-smarter machines, perhaps the wisest path forward is recalling what it is to be human.

## References

[B1] Aizenberg E. Dennis M. J. Van Den Hoven J. (2025). Examining the assumptions of AI hiring assessments and their impact on job seekers' autonomy over self-representation. AI Soc. 40, 919–927. 10.1007/s00146-023-01783-1 40191164 PMC11968453

[B2] Andreescu F. C. (2025). Existence hacked: meaning, freedom, death, and intimacy in the age of AI. AI Soc. 40, 2881–2893. 10.1007/s00146-024-02052-5

[B3] Annabell T. Rasmussen N. V. (2024). Spotify (Un)wrapped: how ordinary users critically reflect on Spotify's datafication of the self within creative workshops. J. Gend. Stud. 1–17. 10.1080/09589236.2024.2433674

[B4] Banja J. (2020). Obviously you, maybe you, artificial you: exploring the impact of artificial intelligence technologies on consciousness and personal identity. AJOB Neurosci. 11, 128–130. 10.1080/21507740.2020.1740349

[B5] Bartley T. (2019). The digital surveillance society. Contemp. Sociol. J. Rev. 48, 622–627. 10.1177/0094306119880195c

[B6] Bauer E. Greiff S. Graesser A. C. Scheiter K. Sailer M. (2025). Looking beyond the hype: understanding the effects of AI on learning. Educ. Psychol. Rev. 37:45. 10.1007/s10648-025-10020-8

[B7] Beatty C. Malik T. Meheli S. Sinha C. (2022). Evaluating the therapeutic alliance with a free-text CBT conversational agent (Wysa): a mixed-methods study. Front. Digit. Health 4:847991. 10.3389/fdgth.2022.847991 35480848 PMC9035685

[B8] Boff Medeiros N. Fogliatto F. S. Karla Rocha M. Tortorella G. L. (2025). Predicting the length-of-stay of pediatric patients using machine learning algorithms. Int. J. Prod. Res. 63, 483–496. 10.1080/00207543.2023.2235029

[B9] Boucher E. M. Harake N. R. Ward H. E. Stoeckl S. E. Vargas J. Minkel J.. (2021). Artificially intelligent chatbots in digital mental health interventions: a review. Expert Rev. Med. Devices 18, 37–49. 10.1080/17434440.2021.2013200 34872429

[B10] Brubaker R. (2020). Digital hyperconnectivity and the self. Theory Soc. 49, 771–801. 10.1007/s11186-020-09405-1

[B11] Calvo R. Peters D. Vold K. Ryan R. (2020). “Supporting Human autonomy in AI systems: a framework for ethical enquiry,” in Ethics of Digital Well-Being. Philosophical Studies Series, eds. C. Burr and L. Floridi (Cham: Springer International Publishing), 31–54. 10.1007/978-3-030-50585-1_2

[B12] Canbul Yaroglu A. (2024). Who's in the mirror: shaping organizational identity through artificial intelligence and symbolic interactionism. Kybernetes. 10.1108/K-09-2024-2379. [Epub ahead of print].

[B13] Chen S. Rui L. Gao Z. Yang Y. Qiu X. Guo S. (2025a). Service migration with edge collaboration: multi-agent deep reinforcement learning approach combined with user preference adaptation. Fut. Gener. Comput. Syst. 165:107612. 10.1016/j.future.2024.107612

[B14] Chen Z. Gan W. Wu J. Hu K. Lin H. (2025b). Data scarcity in recommendation systems: a survey. ACM Trans. Recomm. Syst. 3, 1–31. 10.1145/3639063

[B15] Ciszek E. Price S. Mocarski R. (2025). Identity as epistemology: how transgender and gender-diverse practitioners transform public relations. J. Public Relat. Res. 1–16. 10.1080/1062726X.2025.2506442

[B16] Currie N. K. Wilkinson K. McGeown S. (2025). Reading fiction and psychological well-being during older adulthood: positive affect, connection and personal growth. Read. Res. Q. 60:e605. 10.1002/rrq.605

[B17] Darcy A. Beaudette A. Chiauzzi E. Daniels J. Goodwin K. Mariano T. Y.. (2023). Anatomy of a Woebot^®^ (WB001): agent guided CBT for women with postpartum depression. Expert Rev. Med. Devices 20, 1035–1049. 10.1080/17434440.2023.2280686 37938145

[B18] Dinesh D. N. Rao M. N. Sinha C. (2024). Language adaptations of mental health interventions: user interaction comparisons with an AI-enabled conversational agent (Wysa) in English and Spanish. Digit. Health 10:20552076241255616. 10.1177/20552076241255616 38798884 PMC11119503

[B19] Ferrara E. (2024). Large language models for wearable sensor-based human activity recognition, health monitoring, and behavioral modeling: a survey of early trends, datasets, and challenges. Sensors 24:5045. 10.3390/s24155045 39124092 PMC11314694

[B20] Fesce R. (2024). The emergence of identity, agency and consciousness from the temporal dynamics of neural elaboration. Front. Netw. Physiol. 4:1292388. 10.3389/fnetp.2024.1292388 38628469 PMC11018992

[B21] Good A. P. Horn E. (2025). Unlocking autism's complexity: the move initiative's path to comprehensive motor function analysis. Front. Integr. Neurosci. 18:1496165. 10.3389/fnint.2024.1496165 39906124 PMC11790654

[B22] Inkster B. Kadaba M. Subramanian V. (2023). Understanding the impact of an AI-enabled conversational agent mobile app on users' mental health and wellbeing with a self-reported maternal event: a mixed method real-world data mHealth study. Front. Glob. Womens Health 4:1084302. 10.3389/fgwh.2023.1084302 37332481 PMC10272556

[B23] Kohn P. (2024). “Reflection and self-awareness: cultivating effective leadership mindset,” in Elevating Leadership (Emerald Publishing Limited), 91–118. 10.1108/978-1-83549-564-320241007

[B24] Kumar D. Uchoi E. (2025). Using artificial intelligence for spiritual well-being: conceptualizing predictive models. J. Spiritual. Ment. Health 1–29. 10.1080/19349637.2025.2454427

[B25] Lagerkvist A. Tudor M. Smolicki J. Ess C. M. Eriksson Lundström J. Rogg M. (2024). Body stakes: an existential ethics of care in living with biometrics and AI. AI Soc. 39, 169–181. 10.1007/s00146-022-01550-8

[B26] Lang C. (2024). Dreaming big with little therapy devices: automated therapy from India. Anthropol. Med. 31, 232–249. 10.1080/13648470.2024.2378727 39435587

[B27] Leander K. M. Burriss S. K. (2020). Critical literacy for a posthuman world: when people read, and become, with machines. Br. J. Educ. Technol. 51, 1262–1276. 10.1111/bjet.12924

[B28] Lee M. (2025). Emergence of self-identity in artificial intelligence: a mathematical framework and empirical study with generative large language models. Axioms 14:44. 10.3390/axioms14010044

[B29] Masiero S. (2023). Digital identity as platform-mediated surveillance. Big Data Soc. 10:20539517221135176. 10.1177/2053951722113517635886270

[B30] Mhlambi S. Tiribelli S. (2023). Decolonizing AI ethics: relational autonomy as a means to counter AI harms. Topoi 42, 867–880. 10.1007/s11245-022-09874-2

[B31] Namestiuk S. (2023). Challenging the boundary between self-awareness and self-consciousness in AI from the perspectives of philosophy. Futur. Philos. 2, 43–60. 10.57125/FP.2023.12.30.03

[B32] Reid J. (2024). Digitising “The Big Lie”: algorithmic curation as an inhibitor of media exposure diversity online. Communicatio 50, 1–21. 10.1080/02500167.2024.2424841

[B33] Sackett C. Harper D. Pavez A. (2024). Do we dare use generative AI for mental health? IEEE Spectr. 61, 42–47. 10.1109/MSPEC.2024.10551790

[B34] Schlozman S. Osterberg L. Kassam A. Wolf J. (2025). Developing a framework for mental health disclosure decision-making among medical students: a qualitative pilot study. Am. J. Health Promot. 39, 902–910. 10.1177/08901171251329291 40122671 PMC12144325

[B35] Schueller S. M. Neary M. Lai J. Epstein D. A. (2021). Understanding people's use of and perspectives on mood-tracking apps: interview study. JMIR Ment. Health 8:e29368. 10.2196/29368 34383678 PMC8387890

[B36] Spence R. Ashman R. Patterson A. Hunter-Jones P. (2025). Beyond the body productive: exploring the transformative potential of self-tracking. Sociology 59, 442–465. 10.1177/00380385241297681

[B37] Spytska L. (2025). The use of artificial intelligence in psychotherapy: development of intelligent therapeutic systems. BMC Psychol. 13:175. 10.1186/s40359-025-02491-9 40022267 PMC11871827

[B38] Tan J. H. W. Jayasekara D. N. (2025). Perfect conformity to observable minimal rituals engenders trust: an experimental test of the signaling hypothesis. Appl. Psychol. 74:e12555. 10.1111/apps.12555

[B39] Tan P. (2025). “Disability as innovation and transformation: informing abolitionist elementary mathematics education,” in Handbook for Educating Students with Disabilities, ed. J. P. Bakken (Cham: Springer Nature Switzerland), 1–19. 10.1007/978-3-031-57286-9_35-1

[B40] Turtle G. L. Bendor R. Giaccardi E. Kotowski B. (2024). Queering AI: undoing the self in the algorithmic borderlands. arXiv preprint arXiv:2410.03713. 10.48550/ARXIV.2410.03713

[B41] Wang J. (2023). Self-awareness, a singularity of AI. Philos. Study 13, 68–77. 10.17265/2159-5313/2023.02.003 36820287 PMC9937968

